# Well-Being Benefits of Horticulture-Based Activities for Community Dwelling People with Dementia: A Systematic Review

**DOI:** 10.3390/ijerph191710523

**Published:** 2022-08-24

**Authors:** Theresa L. Scott, Ying-Ling Jao, Kristen Tulloch, Eloise Yates, Oliver Kenward, Nancy A. Pachana

**Affiliations:** 1 School of Psychology, The University of Queensland, St. Lucia, QLD 4072, Australia; 2 College of Nursing, The Pennsylvania State University, University Park, State College, PA 16802, USA; 3 School of Health and Behavioural Sciences, University of the Sunshine Coast, Moreton Bay, Petrie, QLD 4556, Australia

**Keywords:** horticulture, gardening, biophilia, engagement, Alzheimer’s disease, dementia, community, systematic review

## Abstract

Most people living with dementia in the early-to-middle stages live in the community or in their own homes and engagement in enjoyable activities is fundamental to maintaining quality of life and autonomy. Horticulture-based activities are beneficial for the health and well-being for people living with dementia (“PLWD”) in residential care settings, yet evidence within community settings, where the majority live, has not been comprehensively synthesized. A mixed studies systematic review protocol was registered and a systematic search conducted to June 2022 across MEDLINE, COCHRANE, Web of Science, Embase, Psycnet, CINAHL, PsycINFO databases, using terms relating to dementia and horticulture. Original studies examining group or individual horticulture-based programs for community-dwelling PLWD were included. Forty-five articles were selected for full review, eight met inclusion criteria and were retained for data extraction. Evidence from three mixed methods, two quantitative, two qualitative, and one case study design, involving a total of 178 community dwelling PLWD, was narratively summarized. Findings revealed that involvement in horticulture-based activities led to positive impacts on engagement, social interactions, and mental and physical well-being in PLWD. No conclusive evidence was found from included studies for improvement in cognitive function. As most studies to date have concentrated on PLWD in long-term care settings, future research should evaluate the effect of these types of activities in a more rigorous intervention design in community settings.

## 1. Introduction

### 1.1. Background

The global population of people living with dementia is increasing rapidly [1]. The prevalence of dementia is expected to triple from early-century levels by mid-century [2], in turn leading to an increase in the number of people living with dementia (“PLWD”) requiring care [3]. Most PLWD in the early-to-middle stages live in the community, where living at home for as long as possible is not only desirable but linked with increased quality of life [4]. Improving access to meaningful activities and opportunities for engagement is fundamental to maintaining independence, quality of life, and a sense of autonomy and identity [5,6]. Furthermore, a lack of engagement of PLWD is associated with higher caregiver stress and greater likelihood of admission to residential care facilities [7].

The negative impacts of dementia on individuals’ health, well-being, and quality of life can be profound. PLWD are at increased risk of loneliness and isolation [8]. Social isolation and loss of social participation have a substantial negative impact on quality of life, potentially leading to anxiety and depression [9] and more rapid cognitive decline [10], which in turn is associated with early entry to institutional care [11,12]. Conversely, engagement in pleasant activities is a fundamental feature of a good quality of life for persons living with dementia [6,13].

### 1.2. The Need for Non-Pharmacological Intervention for Community-Based Persons with Dementia

Previous research showed that participation in a range of leisure activities was correlated with more preserved cognitive function for people with cognitive decline [14]. Moreover, evidence supports that psychosocial interventions (i.e., non-pharmacological interventions) can help alleviate many of the negative symptoms associated with dementia, such as anxiety, agitation, depression, and apathy [15]. Therefore, there is a critical need to explore suitable non-pharmacological treatments that help slow cognitive decline, relieve the behavioral and psychological symptoms associated with dementia, and consequently improve health outcomes and quality of life for the population of people with dementia living in their own homes [13].

### 1.3. Therapuetic Horticulture

One promising non-pharmacological intervention may be therapeutic horticulture. Therapeutic horticulture describes a process, either active or passive, of purposefully using plants and gardens in therapeutic and rehabilitative activities designed to positively affect a set of defined health outcomes for individuals (e.g., improved mood, improved self-esteem, enhanced social interaction, etc.) [16,17,18]. Therapeutic horticulture-based programs may include individual, community, or group gardening and activities such as watering, weeding, planting, propagating, cultivating, and sensory experiences. Previous studies and systematic reviews have primarily focused on horticulture-based interventions for people with dementia living in nursing homes and custodial dementia care units [19,20,21,22,23,24]. These reviews have revealed benefits of participation, including improved mood, behavior, cognition, and motor function for PLWD in residential care settings. Exposure to sensory stimuli in outdoor activity engagement assisted in the creation of new memories [25].

Additionally, non-pharmacological treatments may be a strong complement to offset some of the psychosocial effects of dementia by leveraging participants’ strengths into new skill sets, facilitating social participation, and providing activities that can be specifically tailored to individuals’ needs and preferences [26]. Such person-centered activities have the further benefit of inclusivity and engagement in meaningful and everyday activities [23]. Therapeutic horticulture activities provide a unique interface of person-centeredness with more biological needs, providing a holistic experience: for example, gardens provide access to fresh air, sunshine, and exercise, which can help regulate circadian rhythms, improve sleep, and control appetite [20]. Regarding people living in institutional care settings, interactions with gardens and horticulture elements can enhance concentration, improve attention and memory, lessen physical pain [27], reduce anxiety, and elevate mood [28]. Communal gardens offered access to social partners for older people without cognitive decline [29], with these social interactions conferring benefits of their own [23,30]. Therefore, horticulture-based activities may be a promising recreation for people with dementia living in the community or in their own homes, who are more often in the early-to-middle stages of the disease, where meaningful activity engagement is important to slow cognitive decline and to sustain independence and quality of life [6].

### 1.4. Biophilia and the Aesthetic Experience of Nature

The biophilia theory [31] provides one theoretical explanation of the therapeutic benefits of nature and gardens and specifically how being in natural environments can contribute to well-being. Wilson [31] used the term biophilia to describe a deep and innate affiliation that humans have with nature as a consequence of evolution. The evolutionary-based theory suggests that we have evolved to prefer and appreciate natural environments because the survival of our species relied on our ancestors’ ability to rest and recover from predators in verdant environments. Ulrich [32] proposed that the human survival advantages of such natural environments were thus genetically encoded as a restorative response to nature. A key concept underpinning the biophilia theory is the aesthetic experience of nature according to Kaplan and Kaplan [33]. That is, we have been genetically programmed to focus on and respond more positively to natural environments and their elements, such as plants, flowers, birds, trees, and water, compared with man-made environments [31,32,33]. Over millions of years of repeated experiences in natural environments encoded humans with a behavioral response (attraction to) and an emotional response (capacity to recover) to natural environments according to the biophilia theory [32]. Evidence in support of the biophilia theory comes from studies that showed participants’ responses to natural environments and their elements included reduced stress, lowered blood pressure, and better immune system functioning [34].

Whereas horticulture-based activities have resulted in improved mood, social interaction, and reduced behavioral symptoms for PLWD in long-term care settings [35], it is also important to understand the benefits for community dwelling persons with dementia, whose needs and experiences differ.

### 1.5. Review Objectives and Research Questions

While previous research has established many benefits of horticulture-based activities for PLWD, the focus of these studies and reviews has been long-term care environments [19,21], mixed populations of people with and without cognitive impairment [36], merely being outdoors [37], in greenspaces [38], or having excluded qualitative findings from PLWD [39]. Murroni et al., (2021), for example, found that garden visits and gardening therapy had some impact on mood and medication use, however the majority of this sample were living in nursing homes. The aim of this review, therefore, was a specific focus on the accumulated evidence for the health and well-being benefits of horticulture-based activities for PLWD in the community. As this population is underrepresented in these previous reviews, our aim was to conduct an up-to-date systematic review and synthesis of the evidence from studies with diverse designs, to guide clinical practice around suitable leisure activities for community dwelling persons with dementia, and to inform future research efforts to strengthen evidence. Our analysis of the extracted data included a focus on the following research questions:

1. What evidence exists about the impact of using horticulture-based activities and interventions to enhance well-being for PLWD in community settings?
2. What is known about the impact of using horticulture-based activities and interventions on behaviors and symptoms associated with dementia?
3. Are there commonalities in evaluation and measurement, i.e., potentially suitable measures and methods that may inform evidence-based practice and future research?

## 2. Methods

### 2.1. Information Sources and Search Strategy

A preliminary search of the International Prospective Register of Systematic Reviews (PROSPERO) database and Cochrane Library was conducted and identified no pre-existing or planned reviews of therapeutic horticulture programs targeted at community-dwelling PLWD. The systematic review followed the checklist from the Preferred Reporting Items for Systematic Reviews and Meta-Analysis (PRISMA) [40] guidelines; the protocol was registered with PROSPERO (CRD42018104497). A search strategy was developed in consultation with a specialist librarian; it included using free text searching of broad terms to capture the greatest number of potentially relevant studies (as shown below) together with relevant Medical Subject Headings (MeSH). Searches were conducted in the COCHRANE Database of Systematic Reviews, Web of Science, MEDLINE, Embase, Psycnet, CINAHL, PsycINFO. A citation chaining of key articles was conducted, searching backward to identify any other published studies, and forward for any new works citing these key articles. The systematic search included all studies up to June 2022.

The following search terms were used:

- Dementia OR Alzheimer disease OR cognitive impairment;
- AND “Respite care” OR “Respite Community Health” OR “Community Health Service” OR “Home Care” OR “Day care” OR “Domiciliary Care” OR Domiciliary OR “Home Care” OR “Home” OR “Home Service” OR “Independent living” OR “Aging in Place” OR “Community Dwelling” OR “Community” OR Dwelling* OR Community OR Living OR Independent;
- AND “Horticultural therapy” OR “Recreational Therapy” OR horticult* OR “Horticultural Therapy” OR “Nature assisted therapy” OR “green care” OR garden* OR “nature assisted” OR “sociohorticultur” OR “ecotherapy” OR “nature based”.

### 2.2. Study Eligibility

#### 2.2.1. Inclusion Criteria

The study eligibility criteria are shown in Table 1. Studies were included if they used recognized methods of qualitative and quantitative data collection and analyses (e.g., survey, interview, observation) focused on horticulture-based activities or interventions via group or individual programs. We included studies if their participants were people residing in the community with any type of dementia and with or without care partner or family member involvement.

#### 2.2.2. Intervention/Activity

The review included interventions and various forms of activity related to horticulture designed to improve or maximize an individual’s health and well-being, from hands-on propagation, garden maintenance, weeding, raking, to more passive involvement such as potting up seedlings, watering, or exploring a garden. Additionally, studies which investigated benefits or effects of involvement in horticulture-based group activities or social gardening programs were considered eligible for this review, such as community gardening.

#### 2.2.3. Exclusion Criteria

We excluded green care farms, since the care and activities provided differ greatly, including agricultural activities, animal interactions, and nursing home care. Studies that focused on people living in nursing homes and residential care settings and those that focused exclusively on care partners and not people living with dementia were also excluded. Studies that focused exclusively (on patients with) delirium were excluded because the time course and patterns of delirium differ from dementia. For example, if treated, delirium may be reversed, unlike dementia. Non-English language publications were excluded. Books, book chapters, dissertations, editorials, review articles, commentaries, opinions pieces, and unpublished grey literature were not included.

#### 2.2.4. Quality Appraisal

Studies that met the inclusion criteria were appraised by two independent reviewers for methodological quality prior to inclusion in the review using a standardized critical appraisal instrument to evaluate the quality of the included studies. Any disagreements that arose between the reviewers were resolved through recourse to a third reviewer. Study quality assessment was based on the Mixed Methods Appraisal Tool (MMAT) version 2018 [41] to assesses the risk of bias across several criteria for systematic mixed studies reviews. For each included study, a category of questions (e.g., qualitative, quantitative, RCT, non-RCT, mixed, etc.) were applied and a number of quality criteria were critically appraised by a minimum of two raters. An overall score was not used, rather the MMAT provided a more detailed appraisal of the studies to inform judgements about their quality.

#### 2.2.5. Screening and Data Extraction

All retrieved studies were exported into the EndNote reference management software and duplicates were removed. An initial screening of all titles and abstracts to identify potentially relevant papers was conducted independently by two reviewers (E.Y. and T.L.S.). Any differences in decisions around potential full text inclusions were reconciled through discussion after all abstracts had been screened. Papers selected for full-text retrieval were first screened for congruence with the review’s inclusion criteria. A data extraction template, constructed to identify specific activities undertaken, the domains assessed, the methods of assessment, and the main findings, was used to extract information from the studies that were retrieved for full review by two reviewers. Table 2 includes this summary information.

#### 2.2.6. Synthesis

We planned a narrative approach to presenting the results, given a high degree of variation in research designs and heterogeneity in the research outcomes and measures across the retrieved studies, making meta-analysis problematic.

## 3. Results

**Figure 1:**
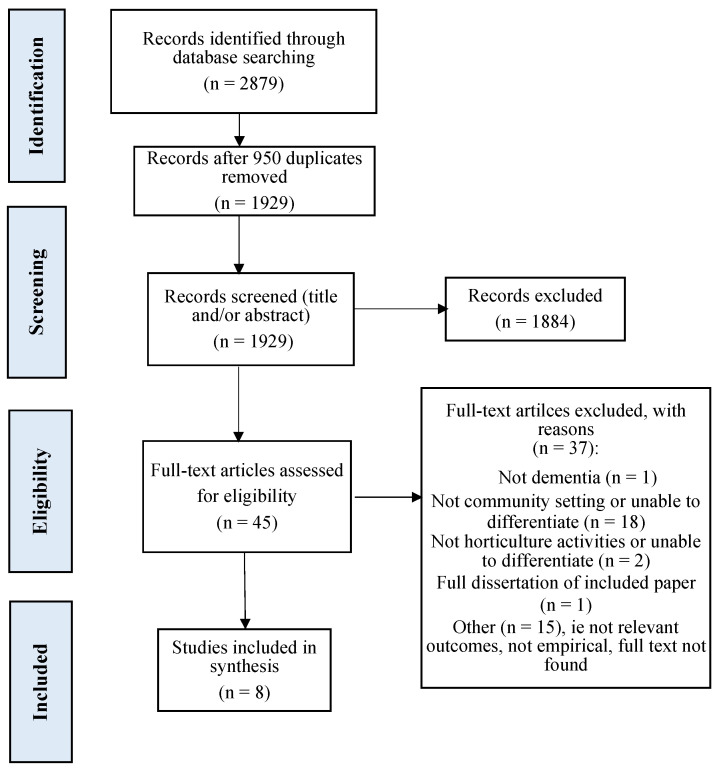
PRISMA flow diagram [40] for selection of papers.

A total of 2879 records were retrieved through database searching and 1929 records for screening after duplicates were removed. Following title and abstract screening, 45 articles remained for full text review. A total of 37 articles were excluded because they did not meet the inclusion criteria as shown in the Figure 1 PRISMA flow diagram. Eight studies met all criteria. Details of these eight included studies are shown in Table 2.

### 3.1. Overview of Studies

Table 2 summarizes the key characteristics for all included studies. The eight studies included two qualitative, two quantitative, one descriptive case study, and three mixed methods studies. Study participants included 178 people living with various types of dementia. Study samples ranged from 6 to 89 participants. The majority of studies included fewer than 14 participants. Seven of eight studies focused on older PLWD, one study specifically recruited people with young-onset dementia [42]. Horticulture-based interventions included two structured horticulture therapy programs, (handcraft with flowers, reminiscence about gardens, planting seedlings), one individualized gardening program (watering, planting, harvesting), and five group based programs (community gardening, digging, planting, maintenance). These eight studies were heterogeneous in terms of the outcomes and measurements.

### 3.2. Study Quality

Quality appraisal was performed by two raters (T.S. and Y.L.J.) using the MMAT [41]. The risk of bias could be considered low to moderate for all eight included studies, which were subsequently retained in the review. The results of quality appraisals for each study are detailed in the Supplementary Table S1.

### 3.3. Outcome Domains

The outcomes for each of the eight studies are shown in Table 2. These domains were diverse, ranging from participants’ engagement in the activities to social interactions, well-being, quality of life, mood, functional performance, memory, and lived experiences. The foremost outcomes included effect of the program or activity on participants’ engagement, well-being, and functional performance levels.

### 3.4. Measurement

Outcome measurements employed across the eight studies varied widely. The diversity of these makes it more difficult to present a meaningful summary of potentially suitable measures to inform evidence-based practice and future research. However, five studies included at least one standardized measurement instrument. The change in cognitive function pre- to post-intervention was measured in one study [42] using the MMSE [43]. Depressive symptoms and memory performance were measured in one study [44] using the Geriatric Depression Scale-15 [45] and Wechsler Memory Scale-Revised [46], respectively. Observation methods were used alongside a rating scale in four studies [47,48,49,50], i.e., the Five-item Observed Emotion Rating Scale [51], an observation tool for rating positive and negative emotions, the Dementia Care Mapping tool [52], an observation measure of quality of life, or the Modified Activity-in-Context [53].

### 3.5. Program Design and Dosage

Program design and dosage varied across studies, however the range of activities included in the programs were largely outdoor based. These activities included a mix of active (digging, planting, harvesting, and sowing seeds) and passive engagements (watering plants and nature walks), which did not systematically vary according to participants’ age. However, older persons were the primary target of recruitment across all studies, with the exception of one study [42]. This one study was a structured gardening program around active engagement, such as digging, and planting, and specifically targeted at people with young-onset dementia who were physically active [42]. Programs that targeted older people living with dementia generally allowed for self-selection from a range of structured and unstructured activities, for example, community gardening sessions where participants could be digging, planting, harvesting, clearing up [44,48,50,54], planting seeds [44,50] or making bird feeders [55]. The more passive engagements included cooking and craft in a garden setting [47,49].

In terms of intervention dosage, the time spent in activities and the frequency of participation across the studies ranged from thirty minutes to two hours a day, one to three days per week, for a total of six weeks to 12 months. Structured horticulture-based activity programs at daycare facilities were time limited as the included studies were more often from pilot trials; these were less frequent and of shorter durations and generally participation ranged from 45–60 min duration and across a period of 6 weeks to 5 months.

### 3.6. Program Outcomes

#### 3.6.1. Cognitive Function

Two of eight studies evaluated the effect of the intervention on cognitive function [42,44]. Hewitt and colleagues [42] conducted a structured community gardening program focused on matching tasks to the ability of the participants, (*N* = 12) living with young-onset dementia. Cognitive functioning measured by MMSE scores declined pre- to post-test over the one-year measurement period (*t*(5) = 3.88, *p* = 0.012), while well-being increased. Makizako and colleagues [44] measured the effect of a horticulture-based intervention on cognitive function, excluding memory, using the verbal fluency test [56] and tablet versions of the trail-making test [57]. Measurements were taken at baseline, as well as post-intervention follow-ups at 6 and 12 months. There were no significant differences in cognitive function between baseline and post-intervention follow-ups.

**Table 2:** Summary information of included studies.

Study	Author(s), Year, Country	Aims	Design	Participants	Intervention or Activity and Intensity	Outcomes	Methods of Data Collection	Main Findings
[47]	Hall et al., (2016) Canada	To examine if and how to increase engagement in horticultural activities through an improved garden design and person-centered recreational programming, compared with more traditional day program activities.	Mixed methods approach. Descriptive analysis and validated observational tool.	A total of 14 participants in early-to-moderate stages of dementia, i.e., diagnosis or suspected diagnosis of dementia, MMSE 16–26, and prior interest in gardening. *M* = 84 years; 28.6% female.	A structured horticultural therapy program at adult daycare program, 2 times per week for 10 weeks. Activities included herb garden tour, educational presentations, pet therapy or music therapy in the garden, shelling peas, planting seeds, watering plants, cleaning up the garden, etc.	Well-being and engagement. Lasting impacts of engagement in horticultural therapy program.	‘Dementia care mapping’ tool [52], assessed based on observations of participants every 5 min during the program. Questionnaire on lasting impact of the intervention completed by the care partners at the end of 10-week program.	For 77.42% of the time, participants had high well-being and engagement with the horticultural therapy. Participants talked more about their experiences in the garden club, expressed happy and enthusiastic emotion, and viewed their gardening work as their personal accomplishment. Four themes identified from qualitative data: Combining structured and unstructured activities. Importance of teamwork. Garden reminiscence. Positive risk taking.
[48]	Hendriks et al., (2016) Netherlands	To develop an approach and decisional tool for personalized nature activities for PLWD.	Mixed methods. Phases 1 and 2: qualitative descriptive design. Phase 3: qualitative descriptive pilot one-group design; thematic and descriptive statistical analysis.	A total of 34 participants across study 3 phases.	Individual and personalized nature activity, e.g., nature walk or a gardening activity, e.g., sowing, watering, fertilizing, harvesting, and cooking with home-grown vegetables. Pilot study executed in Spring 2015; duration of activity engagement recommended as 45–60 min.	Phase 1: preferred or important aspects or activities in nature or outdoor spaces for PLWD. Phase 2: n/a, tool development. Phase 3: (a) behaviors and mood dysregulation and (b) feasibility of personalized nature activities.	Phases 1 and 2: focus group. Phase 3: semi-structured interviews. Observed Emotion Rating Scale [51], Interact instrument (Dorset HealthCare NHS Trust).	Eight themes emerged when being in nature: pleasure; relaxation; feeling fit; enjoying the beauty of nature; feeling free; the social aspect of nature; feeling useful; memories. Preferred activities: walking, cycling, swimming, exercising, sitting outdoors, watching and talking about nature, activities involving animals, flowers or plants, watching films about nature or handcraft with flowers. The decision tools for designing person-centered activities are considered highly to reasonably feasible.
[42]	Hewitt et al., (2013) U.K.	Explore changes in well-being resulting from participation in a structured group gardening program for people with young-onset dementia.	Mixed methods qualitative and quantitative; pretest and posttest.	A total of 12 people with young-onset dementia. Inclusion: confirmed diagnosis of dementia, being physically active and interested in gardening, having a caregiver available, access to transport to attend the program.	Structured group gardening program, 2 h weekly for 46 sessions, across one year. Participants helped plan session activities, which included digging and planting with spring flowering bulbs, sweeping leaves, etc.	Cognitive level, activity participation, daily living activities, well-being.	MMSE [43] completed at baseline and again at study mid- and end-points; Bradford Wellbeing Profile [52] by staff observations; semi-structured interviews with care partners’ pretest and posttest.	Results of MMSE and the Well-being Profile showed increased well-being for participants, despite cognitive functioning continuing to decline over the one-year period. Caregivers reported that participants displayed a renewed sense of purpose, independence, and self-esteem.
[49]	Jarrott et al., (2002) USA	To compare horticultural activity program to usual activities such as games and crafts.	Quasi-experimental design.	Nine community-dwelling PLWD attending an adult day service.	Horticultural therapy program activities, three times per week across 10 weeks; sessions were 30–45 min. Activities were mostly outside, planting, tending to plants and seedlings.	Activity engagement and affect.	Observational, coding for activity and affect. Affect scale adapted from the Dementia Care Mapping tool [52].	Participants significantly more engaged during the horticultural activity sessions compared with usual activities. Affect indicated ‘moderate well-being’, although not statistically different between activity conditions.
[50]	Lassell et al., (2021) USA	To explore quality of life indicators after engagement in adaptive gardening compared with adaptive horse riding for PLWD.	Descriptive case study design.	Eight participants in early-to-moderate stages of dementia; 4 persons self-selected into the community-based adaptive gardening condition, aged 60–98 years (M = 82).	Weekly, one hour-long community-based gardening for eight weeks, compared with an adaptive (horse) riding program. Activities included planting, harvesting, weeding, exploring the garden.	Quality of life indicators: participation and apparent affect (e.g., anxiety, fear pleasure, interest, gaze).	Modified Activity-in-Context-in-Time, observational tool [53] used to code total of 31 h of videotaped data	Both activities supported ‘positive’ and ‘neutral’ quality of life indicators. Riding provided more opportunities for complex activities compared with gardening (*U* = 15, *p* = 0.057), however gardening provided a range of adaptations from more relaxed (e.g., reminiscing, watering) to more active participation (weeding).
[57]	Makizako et al., (2019) Japan	To compare the efficacy of physical exercise, horticulture activities, and control condition.	Single blind RCT	A total of 89 participants with depressive symptoms and mild-memory decline, across 3 conditions: horticulture activities, exercise, control.	Weekly, 60–90 min horticulture-based activity program for 20 weeks. Cultivating, growing, harvesting, group gardening, e.g., planting flowers in public gardens. Exercise included dual-task physical and cognitive, e.g., simple calculation tasks while performing stepping exercises. Control group, education classes involved two 90-min classes, topics, e.g., traffic safety, disaster prevention.	Primary outcome(s) measures, depressive symptoms (Geriatric Depression Scale, GDS-15) [45], and memory performance (Wechsler Memory Scale-Revised) [46].	Three groups compared at baseline, 6 months post-intervention, and 12 months follow-up. Physical performance, social network (Lubben Social Network Scale (LSNS-6) [58], life space, daily physical activity levels (triaxial accelerometer).	GDS-15 scores showed no significant improvements across all groups. Exercise group only obtained higher immediate and delayed recall logical memory scores. Horticulture activity did not improve memory function. Horticulture and control groups showed no differences.
[55]	Noone and Jenkins (2018) UK, Glasgow, Scotland	Exploration of the subjective experience of community-based gardening focused on first person experiences (participants with dementia) and caregivers’ views.	Qualitative design, action research approach.	Six participants with diagnosis of any type of dementia (disease stage not specified) recruited from a day center and three program staff.	Community gardening sessions held once per week for six weeks. Activities included planting seeds and seedlings, making bird feeders.	Qualitative thematic analysis of participants’ experiences attending community-based gardening program and views of day center staff.	Semi-structed group interviews with participants and individual interviews with three staff following each of the gardening sessions; researcher reflections.	Gardening is an articulation of identity and selfhood and an expression of agency. It also helps develop new social bonds based on shared interests. Three themes to emerge from interviews were: (i) identity, (ii) agency, (iii) community.
[54]	Smith-Carrier et al., (2019) Canada	To explore emotional and sensory experiences of therapeutic gardening for persons with dementia.	Qualitative design.	Six persons with early-stage dementia attending an adult day center.	Therapeutic group gardening program, six waves across five months of spring and fall activities, e.g., planting, harvesting, pruning, clean-up, etc.	Interpretive analysis of participants’ reflections on sensory and emotional experiences in the gardening process.	Six repeated interviews with participants with dementia.	Themes derived from analysis of interviews provided support for the value of gardening for activating the senses, meaningful occupation, socialization, and mental and physical well-being.

#### 3.6.2. Memory

One study included in this review measured the effect of a horticulture-based activity program on memory performance [44]. Participants (*N* = 89) with mild memory decline were randomly assigned to horticulture, exercise, or control groups. Memory performance was measured using the Wechsler Memory Scale-Revised [46] at baseline and 6 and 12 months post-intervention. The horticulture activities intervention group showed no significant difference in memory performance when compared with the control at 6 and 12 month follow-ups.

#### 3.6.3. Physical Function

The effects of a horticulture activity intervention on physical function were measured in one study [44]. Physical function was measured using normal walking speed and two-minute walking tests. For participants in the horticulture activity intervention group, there was no significant difference in functional performance outcomes when comparing pre- and post-intervention scores.

#### 3.6.4. Social Interaction

Five studies reported social interaction or socializing as an outcome of program participation [44,47,50,54,55]. ‘Teamwork’ emerged as a theme in a 10-week structured horticulture activity program [47], assessed by means of the Dementia Care Mapping tool [52]. Participants were observed working together toward certain goals and having common objectives and experiences, which promoted shared communication about the horticulture activity program and about their experience of living with dementia [47].

Community-based gardening held indoors and outdoors helped to develop new social bonds for six participants with dementia who were part of a larger group of day center attendees [55]. The garden was located in the grounds of a community hall where participants attended a day center and were involved in designing the gardening sessions. Activities included learning about and planting fruit and vegetable seeds and seedlings and ornamentals in hanging baskets. Although the members of the gardening group were known to each other through their membership to the larger day center group, the results revealed that a shared interest in gardening helped develop a new and positive group dynamic, which was distinct from the larger group [55]. Smith-Carrier and colleagues [54] conducted a qualitative investigation of six participants attending a therapeutic gardening program at an outdoor garden attached to an adult day center. Results showed that gardening in a group created a shared sense of identity and enabled a connection to others and the development of intimate relationships [54]. Lassell and colleagues [50] found a group-based horticulture activity intervention appeared to support social interaction amongst participants [50]. One study [44] found no significant difference in pre- and post-intervention scores on the self-report Lubben Social Network Scale 6 [58], subjectively measuring participants’ social connections.

#### 3.6.5. Well-Being and Quality of Life

Six of the included studies reported that participants’ well-being improved after intervention. The majority of these studies were qualitative, with the exception of two studies. The first, a 10-week structured horticulture activity program resulted in high levels of observed well-being for 77.42% of the time that participants were engaged in horticulture activities and these high levels were sustained after program completion according to care partners [47]. The second, a community gardening program targeted at participants with young-onset dementia led to increased mean well-being scores across the first eight sessions for participants, which leveled off as scores reached ceiling levels, however these did not reach significance [42]. One qualitative descriptive study discovered eight themes from focus group discussions with PLWD: pleasure, relaxation, feeling fit, enjoying the beauty of nature, feeling free, social aspect of nature, feeling useful, and memories [48]. A descriptive case study aimed to assess the effect of a horticulture activity or a horse-riding intervention on quality of life. This was done by filming participant activity during interventions and subsequently coding behaviors. Participants in the horticulture activity intervention were seen to express emotional well-being through either interest, pleasure, or engagement with the environment [50]. An earlier study used a similar design, coding participant activity and affect while completing horticulture-based tasks or non-horticulture tasks. While there was no significant difference in affect between groups, participants exhibited moderate levels of well-being throughout the intervention [49]. A single blind randomized control trial measuring the impact of horticulture and exercise interventions on a range of variables, assessed health-related quality of life pre- and post-intervention using the Short Form Health Survey-12 [59]. The horticulture group showed no significant change in health-related quality of life at post-intervention or follow-up assessments [44]. Finally, Smith-Carrier and colleagues [54] found that being actively engaged outdoors led to participants’ self-reported positive appraisals of their physical and emotional well-being.

## 4. Discussion

The aim of this review was to identify research studies that evaluated well-being benefits of horticulture-based activities for community-dwelling people living with dementia. Additionally, we aimed to examine whether using horticulture-based activities positively impacted the behaviors and symptoms associated with dementia. Given that only eight studies were included, it highlights the narrow extent of research that has been conducted in the community setting to date. However, of the eight studies that our search strategy found, many reported benefits of involvement in gardening and therapeutic horticulture programs and none reported any adverse events. We conclude by discussing the limitations of the included studies as related to implications for future research and evidence-based practice.

The analysis of our first question revealed benefits of horticulture-based activities and interventions, including engagement and increases in activity [48] and promoting social participation [47,50,54,55]. Moreover, engaging in gardening and horticulture-related activities had positive implications for identity [55], self-worth [54], sense of purpose and meaning-making [42,47,48], and general well-being [47,49,50]. Given a lack of quantitative indicators or the failure to reach significance levels across the included studies, further high-quality research using more robust clinically meaningful measures in community settings is necessary. For example, Smith-Carrier and colleagues [54] found that participants reported a reduction in the perception of certain physiological symptoms such as pain, but no measurements of functional performance or medical conditions were included. In other research contexts however, significant improvements in physiological outcomes are reported. Pedrinolla et al. [60] conducted a single-blind randomized controlled trial with hospital patients living with dementia and found an indoor therapeutic garden achieved a significant reduction in blood pressure, salivary cortisol, quetiapine dosage, and scores on the Neuropsychiatric Inventory [61], while also improving scores on the Mini-Mental State Examination [43], compared with a control therapy.

### 4.1. Key Features of Programs Promoting Well-Being

Programs that successfully showed well-being improvements for people living with dementia in the community had several features in common. They provided a range of activities in which participants could engage, allowing for customization to suit individual preferences and abilities. Such activities might include potting, weeding, watering, touching, smelling, planning garden beds, harvesting fruit, vegetables, and herbs, and food preparation. Programs were often structured, providing consistency and routine for those living with dementia. Such structure can be important for sustaining activity levels [62], reducing agitation and negative affects [63], and may help individuals retain a sense of identity independent of their dementia status [64]. Earlier research has highlighted the importance of ensuring that structure is developed for the program participant’s benefit, tailoring programs and activities to meet the individual’s needs and capacity at all stages of the disease, not for the benefit of the community service providers or staff [65]. The degree to which the present programs were structured from a person-centered perspective is not consistently reported and should be included in future research.

The programs reviewed here were person-centered in other ways, such as providing emphasis on the ownership or purpose for individuals by allocating a specific plot to each participant [47]. This gave not only a sense of purpose for the individual responsible for a given plot, but also an opportunity to articulate identity or selfhood through expression of agency. An individual may have had degrees of creative control over how they chose to develop their plot [47], their own tasks to complete [48,50], or input into session planning tailored to individual abilities [42]. This is consistent with findings that individuals who can engage in personally meaningful or individualized activities experience reduced agitation and increased pleasure and interest [66]. However, Travers and colleagues [66] identified that this may be an effect of therapies being administered in a one-on-one format rather than a feature of the therapy itself, which remains an area for exploration in horticultural therapy. In the study by Hall et al. [47], the program’s group format facilitated shared responsibilities and opportunity for community contributions, whereby other participating gardeners living with dementia stepped in if an individual was absent.

Programs that address community contributions and shared responsibilities may further aid in alleviating issues related to social isolation, which is increasingly acknowledged as a major barrier to living well with dementia. Group gardening programs that provide opportunities to collaborate around a shared identity are likely to foster feelings of connection and belonging [67]. Elements of community spirit were common across studies, whereby social participation was promoted alongside meaningful activity in terms of planting, cultivating, and harvesting. These activities provide engagement that can build on authentic interactions of goal setting and sharing and finding common interests based on past experiences. This was demonstrated in several of the studies reviewed here, including a structured group gardening program for people with young-onset dementia [42] and community gardening that promoted social citizenship, expression of self-hood, and agency [55].

In addition to the social benefits of the gardening programs reviewed here, there is the further benefit of engagement in activities not just for the sake of it, as may be found in short-term one-off activities in daycare centers that are unrelated to residents’ existing sense of self, such as games and puzzles. Medium-term projects such as gardening may be of particular value for individuals living with dementia in the community, as they foster a sense of longevity and working towards something meaningful, possibly something that will transcend the individual and provide a legacy, a concept that contributes to *hope* in terminal illness [68]. In addition to providing an opportunity to express one’s identity, gardening programs may offer the individual a sense of maintaining that identity [37] and growing [69].

### 4.2. Alleviating Behaviors and Symptoms Associated with Dementia

Regarding the second question, no study specifically measured potential changes in behavioral and psychological symptoms of dementia as an outcome of participation. However, the results of included studies suggest the potential for therapeutic horticulture-based interventions as a suitable non-pharmacological approach to diminishing episodes of distress, in particular as they relate to apathy through increased activity engagement [49,50,54] and reducing feelings of depression and increasing levels of happiness [42,44,54]. The biophilia theory argues that there is a genetic component to the aesthetic response to gardens, linked to our evolutionary experience, which includes the supposition that humans intuitively view natural environments as being places more likely to provide tranquility, security, and shelter [31,32,33,34].

In contrast to some non-pharmacological interventions, horticulture-based programs may be widely accepted because they draw on individuals’ affinity with the natural world [31]. One of the key concepts underpinning the biophilia theory is the aesthetic experience of nature. Moreover, gardens and nature featuring in the daily lives of community-dwelling PLWD, such as their yards or local parks, are part of everyday home making [70], and gardening is a commonly reported leisure activity, in particular for older people [71]. Further, garden-based activities are uniquely placed to be particularly forgiving of changes in ability or absences. Gardening can be a short-, medium-, or long-term activity, providing the benefit of sessions that can be completed individually or as a series. The garden continues to grow in the participant’s absence, providing a sense of continuity and progress, and a high degree of success can be achieved without a great deal of precision. In contrast, skillful and precise craft-based activities such as knitting may highlight errors as a source of frustration to participants.

### 4.3. Implications for Future Research

Investigating our third question about commonalities in evaluation and measurement, the present systematic review highlights several knowledge gaps in the area. Firstly, there is a relative lack of controlled trials and standardized measures, and sample sizes were often small. The voices of people living with dementia were only present in half the studies evaluated here [42,48,54,55]. Of these, the majority used focus groups or interviews with people living with dementia; some included tasks or measurements completed by the person living with dementia. This may be an improvement since Gibson et al. [72] commented that the perspectives of those living with dementia had been ignored by research. Of those evaluated here, studies that did not directly ask the participants living with dementia for their perspectives and experiences largely reported data based on observations by research staff [47,49,50], which may be subject to biases. While observation and proxy measures are valuable inclusions when cognitive impairment limits a person’s ability to communicate their perceptions of their own quality of life [73], the inclusion of direct experiences of people living with dementia is essential for understanding the lived experience of dementia and further for providing person-centered care that focuses on individuality and personhood [74]. Similarly, the degree to which any persons living with dementia were consulted in the development of programs or the design of gardens was only reported by two studies [48,55], although Smith-Carrier et al. [54] invited feedback from participants during the gardening process. Action research [55], co-creation, and participatory design [75] have substantial potential in improving the suitability and acceptability of programs for end users in a range of areas. Such approaches allow for the development of programs that are linked with end users’ desired experiences and the specific needs of a local community.

Differences observed in groups that were involved in horticultural therapy versus those that were not lacked control over participant preferences and perceived or actual capabilities. Ageism and self-stigma arising from dementia diagnoses may influence the types of day centers and community services that participants and their families choose, and participants who self-select more active services may have greater self-efficacy and consequently greater outcomes [76,77,78]. Several of the studies reviewed here specifically sought people who already had an interest in gardening (e.g., [42,47]), making it difficult to know whether the results are from the benefits of gardens and the horticulture related activities or from the benefits of engaging in an enjoyable hobby consistent with one’s existing preferences. Further research should aim to offer clarity around these potentially confounding factors.

### 4.4. Limitations of Review

Although several databases were searched and a rigorous cross-referencing method was used, it is possible that some studies may have been missed. For example, only English language studies and studies for which we had access to full manuscripts were included. In addition, the results must be considered in view of the heterogenous nature of the included studies in terms of varying quality, design, and methods of evidence gathering, such as interviews, observations, and standardized scales.

## 5. Conclusions

According to this review, positive effects were reported for psychological, physical, and social outcomes of participation in horticulture-based programs. However, as is often unavoidable in research involving PLWD, several of the included studies were time limited, sample sizes were small and studies were consequently underpowered, and few studies employed a control or comparison group. Notwithstanding that observational measures and proxy reports are valuable inclusions if cognitive impairment limits an individual’s ability to communicate, the direct input from people living with dementia is necessary to understand the desired outcomes of engagement with horticulture-based interventions and gardening programs. That is, further research is required to co-design and evaluate the therapeutic benefits of horticulture-based interventions for people living with dementia in the community.

## Figures and Tables

**Table 1:** Study eligibility criteria.

	Inclusion Criteria	Exclusion Criteria
Types of studies	All study designs that focus on horticulture-based activities or interventions, group, or individual gardening programs.	Editorials, commentaries, opinions pieces, and unpublished grey literature.
Population	People living with dementia or dyads (PLWD and their family members/care partners); age open (living with young- or late-onset dementia included).	Studies that focus exclusively on care partners not included.
Condition	Dementia, all types included.	Delirium not included.
Setting	PLWD in the community.	Studies that focus on PLWD in residential care facilities, assisted living, or nursing homes not included.
Sources	All studies published in peer reviewed journals, empirical, interventions, case studies in the community.	Publications in languages other than English.

## Data Availability

Not applicable.

## Supplementary Materials

The following supporting information can be downloaded at: https://www.mdpi.com/article/10.3390/ijerph191710523/s1, Table S1: Risk of bias for included studies.
