# Supplementary material for: Well-Being Benefits of Horticulture-Based Activities for Community Dwelling People with Dementia: A Systematic Review
Source: Int J Environ Res Public Health. 2022 Aug 24;19(17):10523. doi: 10.3390/ijerph191710523 (PMC9517764; doi:10.3390/ijerph191710523)
Supplement: Supplementary file 1 [file ijerph-19-10523-s001.zip › ijerph-1849715-supplementary.pdf]

Table S1. Quality assessment table.

	Study	Categories of Study Designs	Methodological Quality Criteria	Responses			
				Yes	No	Can't Tell	Comments
[47]	Hall, et al., (2016)<br><br>Canada	Mixed methods	S1. Are there clear research questions?	Yes			
			S2. Do the collected data allow to address the research questions?	Yes			
			1.1. Is the qualitative approach appropriate to answer the research question?	Yes			
			1.2. Are the qualitative data collection methods adequate to address the research question?	Yes			
			1.3. Are the findings adequately derived from the data?	Yes			
			1.4. Is the interpretation of results sufficiently substantiated by data?	Yes			
			1.5. Is there coherence between qualitative data sources, collection, analysis and interpretation?	Yes			
[48]	Hendriks, et al., (2016)<br><br>Netherlands	Mixed methods	S1. Are there clear research questions?	Yes			
			S2. Do the collected data allow to address the research questions?	Yes			
			5.3. Are the outputs of the integration of qualitative and quantitative components adequately interpreted?	Yes			
			5.4. Are divergences and inconsistencies between quantitative and qualitative results adequately addressed?	Yes			
			5.5. Do the different components of the study adhere to the quality criteria of each tradition of the methods involved?	Yes			
[42]	Hewitt, et al., (2013)<br><br>U.K.	Mixed methods	S1. Are there clear research questions?	Yes			
			S2. Do the collected data allow to address the research questions?	Yes			
			1.1. Is the qualitative approach appropriate to answer the research question?	Yes			
			1.2. Are the qualitative data collection methods adequate to address the research question?	Yes			
			1.3. Are the findings adequately derived from the data?	Yes			
			1.4. Is the interpretation of results sufficiently substantiated by data?	Yes			
			1.5. Is there coherence between qualitative data sources, collection, analysis and interpretation?	Yes			
[49]	Jarrott, et al., (2002)<br><br>USA	Quantitative non-randomized	S1. Are there clear research questions?	Yes			
			S2. Do the collected data allow to address the research questions?	Yes			
			4.1. Is the sampling strategy relevant to address the research question?	Yes			
			4.2. Is the sample representative of the target population?	Yes			
			4.3. Are the measurements appropriate?	Yes			
			4.4. Is the risk of nonresponse bias low?	Yes			
			4.5. Is the statistical analysis appropriate to answer the research question?	Yes			

[50]	Lassell , et al., (2021)<br><br>USA	Observational	S1. Are there clear research questions?	Yes			
			S2. Do the collected data allow to address the research questions?	Yes			
			4.1. Is the sampling strategy relevant to address the research question?	Yes			
			4.2. Is the sample representative of the target population?			X	N = 8 people with dementia
			4.3. Are the measurements appropriate?	Yes			
			4.4. Is the risk of nonresponse bias low?	Yes			
			4.5. Is the statistical analysis appropriate to answer the research question?	Yes			
[57]	Makizako, et al., (2019)<br><br>Japan	Quantitative non-randomized	S1. Are there clear research questions?	Yes			
			S2. Do the collected data allow to address the research questions?	Yes			
			4.1. Is the sampling strategy relevant to address the research question?	Yes			
			4.2. Is the sample representative of the target population?	Yes			
			4.3. Are the measurements appropriate?	Yes			
			4.4. Is the risk of nonresponse bias low?	Yes			
			4.5. Is the statistical analysis appropriate to answer the research question?	Yes			
[55]	Noone and Jenkins (2018)<br><br>U.K. Glasgow Scotland	Qualitative	S1. Are there clear research questions?	Yes			
			S2. Do the collected data allow to address the research questions?	Yes			
			4.1. Is the sampling strategy relevant to address the research question?	Yes			
			4.2. Is the sample representative of the target population?	Yes			
			4.3. Are the measurements appropriate?	Yes			
			4.4. Is the risk of nonresponse bias low?	Yes			
			4.5. Is the statistical analysis appropriate to answer the research question?	Yes			
[54]	Smith-Carrier, et al., (2019)<br><br>Canada	Qualitative	S1. Are there clear research questions?	Yes			
			S2. Do the collected data allow to address the research questions?	Yes			
			4.1. Is the sampling strategy relevant to address the research question?	Yes			
			4.2. Is the sample representative of the target population?			X	n = 6 people with dementia
			4.3. Are the measurements appropriate?	Yes			
			4.4. Is the risk of nonresponse bias low?	Yes			
			4.5. Is the statistical analysis appropriate to answer the research question?	Yes			

Note. Risk of Bias assessment based on the MMAT [41].
